# A decade after the first Pucciniales genomes: A bibliometric snapshot of (post) genomics studies in three model rust fungi

**DOI:** 10.3389/fmicb.2022.989580

**Published:** 2022-09-14

**Authors:** Benjamin Petre, Sébastien Duplessis

**Affiliations:** Université de Lorraine, INRAE, IAM, Nancy, France

**Keywords:** fungal parasite, plant disease, transcriptome, basidiomycete, microbe, bibliometric analysis, virulence protein, plant pathology

## Abstract

Pucciniales (rust fungi) are one of the largest fungal order of plant pathogens. They collectively infect key crops such as wheat and soybean, and threaten global food security. In the early 2010s, the genome sequences of three rust fungi were released: *Melampsora larici-populina* (the poplar leaf rust fungus), *Puccinia graminis* f. sp. *tritici* (the wheat stem rust fungus), and *Puccinia striiformis* f. sp. *triciti* (the wheat stripe rust or wheat yellow rust fungus). The availability of those genomes has forwarded rust biology into the post-genomic era, sparking a series of genomics, transcriptomics, *in silico*, and functional studies. Here, we snapshot the last 10 years of post-genomics studies addressing *M. larici-populina*, *P. graminis* f. sp. *tritici*, and/or *P. striiformis* f. sp. *tritici*. This mini-review notably reveals the model species-centered structure of the research community, and highlights the drastic increase of the number of functional studies focused on effectors since 2014, which notably revealed chloroplasts as a central host compartment targeted by rust fungi. This mini-review also discusses genomics-facilitated studies in other rust species, and emerging post-genomic research trends related to fully-phased rust genomes.

## Introduction

Rust fungal pathogens infect key crops and threaten global food security. Despite their importance, the research community studies them to a limited extent, mostly due to technical limitations associated with the lack of reverse genetics tools. Rust fungi belong to the order pucciniales; one of the largest fungal pathogen order (Aime and McTaggart, 2021). Pucciniales can infect a wide variety of host plants, and attract a particular attention in the phytopathology community because they impact major crops worldwide (Moscou and van Esse, 2017). Rust fungi are obligate biotrophs, have a complex lifecycle, and do not infect some model plants; so far, those features have precluded the establishment of routine approaches for reverse genetics and pushed the research community to use heterologous plant systems (Lorrain et al., 2018; Duplessis et al., 2021). In the late 2000s, the rust biology research community acknowledged the necessity to quickly obtain the first rust genome sequences, and joined efforts on key model rust species (Duplessis et al., 2014; Aime et al., 2017).

In the early 2010s, three publications reported the first genome sequences of three rust fungi: *Melampsora larici-populina* (the poplar leaf rust fungus), *Puccinia graminis* f. sp. *tritici* (the wheat stem rust fungus), and *Puccinia striiformis* f. sp. *triciti* (the wheat stripe rust or wheat yellow rust fungus) (Cantu et al., 2011; Duplessis et al., 2011; Zheng et al., 2013). *M. larici-populina* and *P. graminis* f. sp. *tritici* genomes were obtained through first generation sequencing technology (i.e., Sanger sequencing), whereas *P. striiformis* f. sp. *tritici* genome sequences were obtained through either first or second generation sequencing technologies (i.e., Sanger and/or Illumina) (Duplessis et al., 2014). Although these pioneering attempts led to various levels of quality of genome assembly and annotation, they revealed unique features among pucciniales, which has been proven true since then: rust fungi possess large genomes, with a substantial content in repeat elements (e.g., transposable elements) and with expanded gene families and total number of predicted genes (Duplessis et al., 2013; Aime et al., 2017). The availability of those genome sequences has forwarded rust biology into the post-genomic era, sparking a series of studies that addressed fundamental questions related to the evolution, biology, and virulence mechanisms of rust fungi (Figueroa et al., 2016; Lorrain et al., 2019).

This mini review provides readers with an overview of (post) genomic research that addressed *M. larici-populina*, *P. graminis* f. sp. *tritici*, or *P. striiformis* f. sp. *tritici*, by identifying and analyzing 82 rust (post) genomics publications (hereafter “RPGs”) published between 2010 and 2021. We first snapshot the collection of RPGs and associated publication metrics. Then, we show how the first genomics studies facilitated both a wave of functional analyses of candidate effectors proteins (hereafter “effectors”) as well as studies in other rust species. Finally, we discuss emerging research trends in the field of rust biology, which are directly related to genomics.

## A total of 82 RPGs addressed three model rust fungi since the public release of their genome sequences

**Figure 1:**
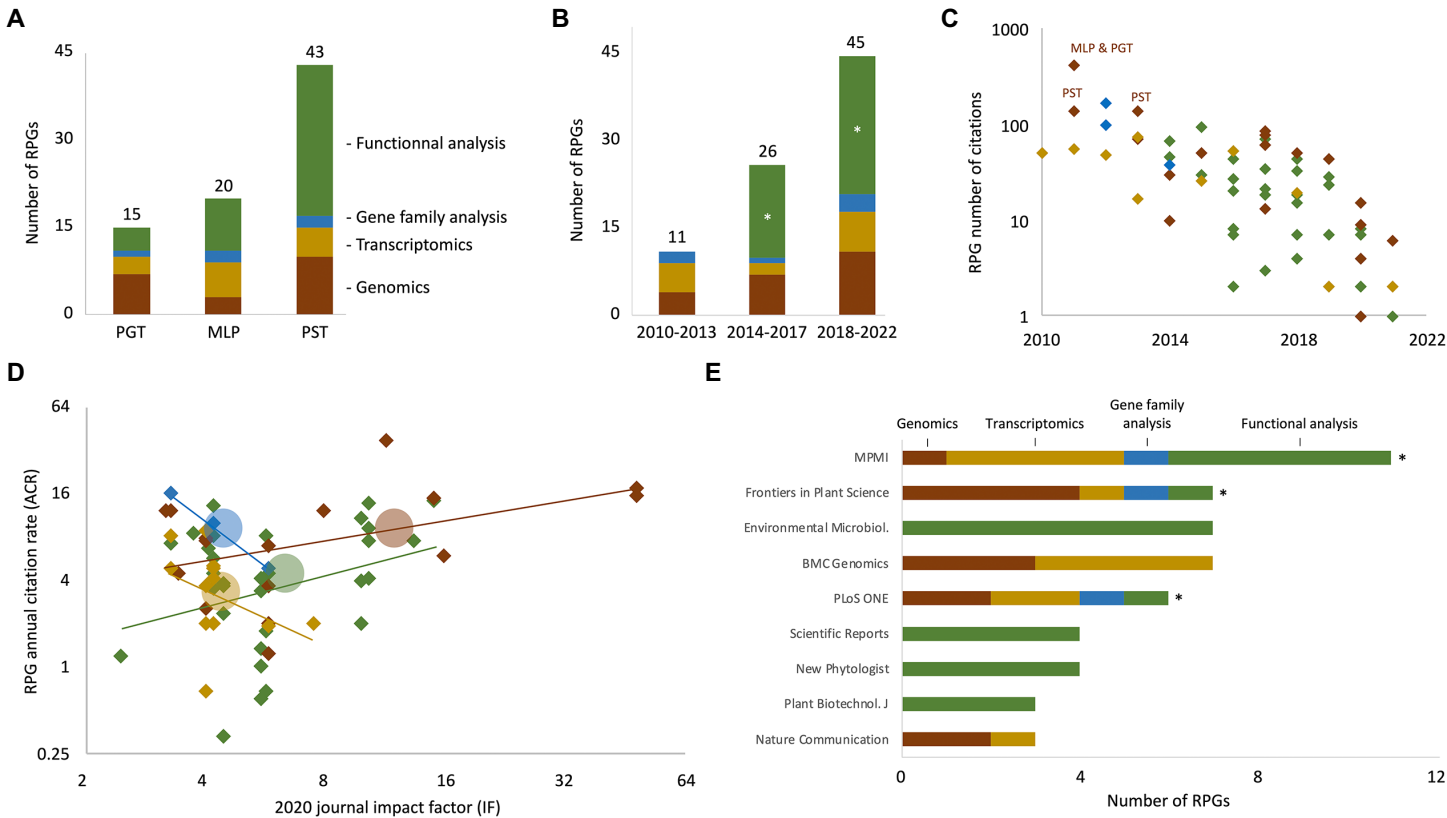
Overview of the 82 rust (post) genomic publications (RPGs) analyzed in this study. **(A)** Bar chart indicating the number of RPGs that primarily report genome analyses (i.e., genomics; brown), transcript profiling analyses (i.e., transcriptomics; yellow), gene family analyses (blue), or functional analyses (green), and which address *Puccinia graminis* f. sp. *tritici* (PGT), *Melampsora larici-populina* (MLP), or *Puccinia striiformis* f. sp. *tritici* (PST). Numbers above each bar indicate the total number of RPGs for each species. The four RPGs that address a combination of *P. graminis* f. sp. *tritici*, *M. larici-populina*, and *P. striiformis* f. sp. *tritici* are not shown. **(B)** Bar chart indicating the evolution of the number of RPGs that pertain to genomics, transcriptomics, gene family, of functional analyses throughout three time periods: 2010–2013, 2014–2017, and 2018–2021; numbers and color code as in panel **A**. The asterisks highlight the “functional analyses” stacks that varied from 0 RPG in 2010–2013 to 15 and 24 RPGs for the periods 2014–2017 and 2018–2022, respectively. **(C)** Scatterplot displaying the number of total citations of individual RPGs according to their year of publication. The total number of citations was extracted from the Web of Science portal in March 2022. Color code as in panel **A**. Thirteen RPGs published in 2021 or 2022, and which have not yet been cited, are not shown on the graph. Acronyms indicate the three RPGs reporting reference genomes in the early 2010s. **(D)** Scatterplot displaying the annual citation rate of individual RPGs according to the value of the 2020 impact factor of the journal in which they were published. Linear trend lines (lines) and average citation rate and impact factor values (circles) are indicated for each of the four categories of RPG. Same color code as in panel **A**. **(E)** Horizontal bar chart indicating the 10 journals that published the most RPGs. Stacks indicate the category (i.e., primary focus) of the RPGs, with the same color code as in panel **A**. Asterisks indicate the three journals that published the four types of studies. Noteworthy, 8 out of the 10 journals adopt a gold open-access model. The raw data used to build this figure are available in Supplementary Dataset 1 (columns A–P).

To identify, collect, and analyze RPGs, we adapted a bibliometric pipeline we recently developed, which mostly uses iterative key word searches on the Web of Science portal (Petre et al., 2022). In total, those searches identified 82 non-redundant RPGs (Supplementary Dataset 1 – columns A–M). Only four RPGs addressed more than one of the three rust model species; the remaining 78 RPGs addressed specifically *P. striiformis* f. sp. *tritici*, *M. larici-populina*, or *P. graminis* f. sp. *tritici*, with 43, 20, and 15 RPGs, respectively (Figure 1A). We assigned each RPG to one of the four following categories, according to its primary finding reported and/or main approach used: (i) “Genomics” (22 RPGs; analysis, re-analysis, or comparison of genome sequences), (ii) “Transcriptomics” (14 RPGs; sRNA or mRNA profiling studies, *in laboratorium* or in the field), (iii) “Gene family analysis” (6 RPGs; *in silico* gene family annotation and analyses), and (iv) “functional analysis” (40 RPGs; studies that functionally characterized specific molecules, mainly effector proteins – see next section). Twenty-nine RPGs reported findings that pertain to more than one category; for instance, seven RPGs from the “Genomics” category also used transcriptomic and gene family analyses. For those 29 RPGs, we used expert reading of the manuscripts to select the most relevant primary category. RPGs from all four categories addressed *M. larici-populina*, *P. graminis* f. sp. *tritici*, and *P. striiformis* f. sp. *tritici*, indicating that all typical (post) genomic approaches are used in these three model species (Figure 1A). The number of RPGs published increased during the last decade, from 11 RPG for the 2010–2013 period to 45 for the 2018–2022 period (Figure 1B). Interestingly, the first “functional analysis” RPGs were published only in 2014, but dominate the share of RPGs published after 2018; by contrast, RPGs from the three other categories were regularly published over the last decade. This suggests that functional investigations of rust specific proteins have emerged and then rapidly increased in number within the 10 years following the sequencing of the first rust fungal genomes.

To better comprehend the collection of RPGs, and notably to better apprehend the publication habits of the research community, we performed a simplified citation analysis (Petre et al., 2022). Firstly, as expected, we observed that RPGs steadily accumulate citations over time (Figure 1C; Supplementary Dataset 1 – columns N–P). On average, RPGs receive approximately 6 citations per year (i.e., annual citation rate of 6). Two of the RPGs with both the highest number of total citations and the highest annual citation rate reported reference genome sequences of *M. larici-populina*, *P. graminis* f. sp. *tritici*, and *P. striiformis* f. sp. *tritici* (Duplessis et al., 2011; Zheng et al., 2013); illustrating the central importance of early genomics studies. Secondly, we observed that annual citation rates of the RPGs of the “Transcriptomics” and “Gene family analysis” negatively correlate with journal impact factors (Figure 1D). Notably, “gene family analysis” RPGs appear in journals with modest impact factors (4.38 on average) but show high annual citation rates (10.2 on average) (Hacquard et al., 2012; Saunders et al., 2012; Sperschneider et al., 2014). Also, 9 out of the 20 RPGs with an annual citation rate superior to 8 appeared in a journal with an impact factor inferior to 5. Altogether, this suggests that journal impact factors poorly predict RPGs success in terms of citation, especially for RPGs reporting gene family and transcriptome analyses. Finally, an analysis of the 10 journals that published the highest number of RPGs revealed “specialized” journals that publish only RPGs from the “genomics” or “transcriptomics” categories (e.g., BMC Genomics) or exclusively “functional analysis” RPGs (e.g., environmental microbiology) (Figure 1E). Interestingly, it also revealed three journals (Molecular Plant-Microbe Interactions, Frontiers in Plant Science, and PLOS ONE) that publish RPGs from all four categories and that address the three rust species; this suggests that those three journals are central in the field of rust biology and may assist information flow between researchers tackling different research questions and objects.

## Pucciniales genomes facilitated the investigation of rust fungal candidate effectors

**Figure 2:**
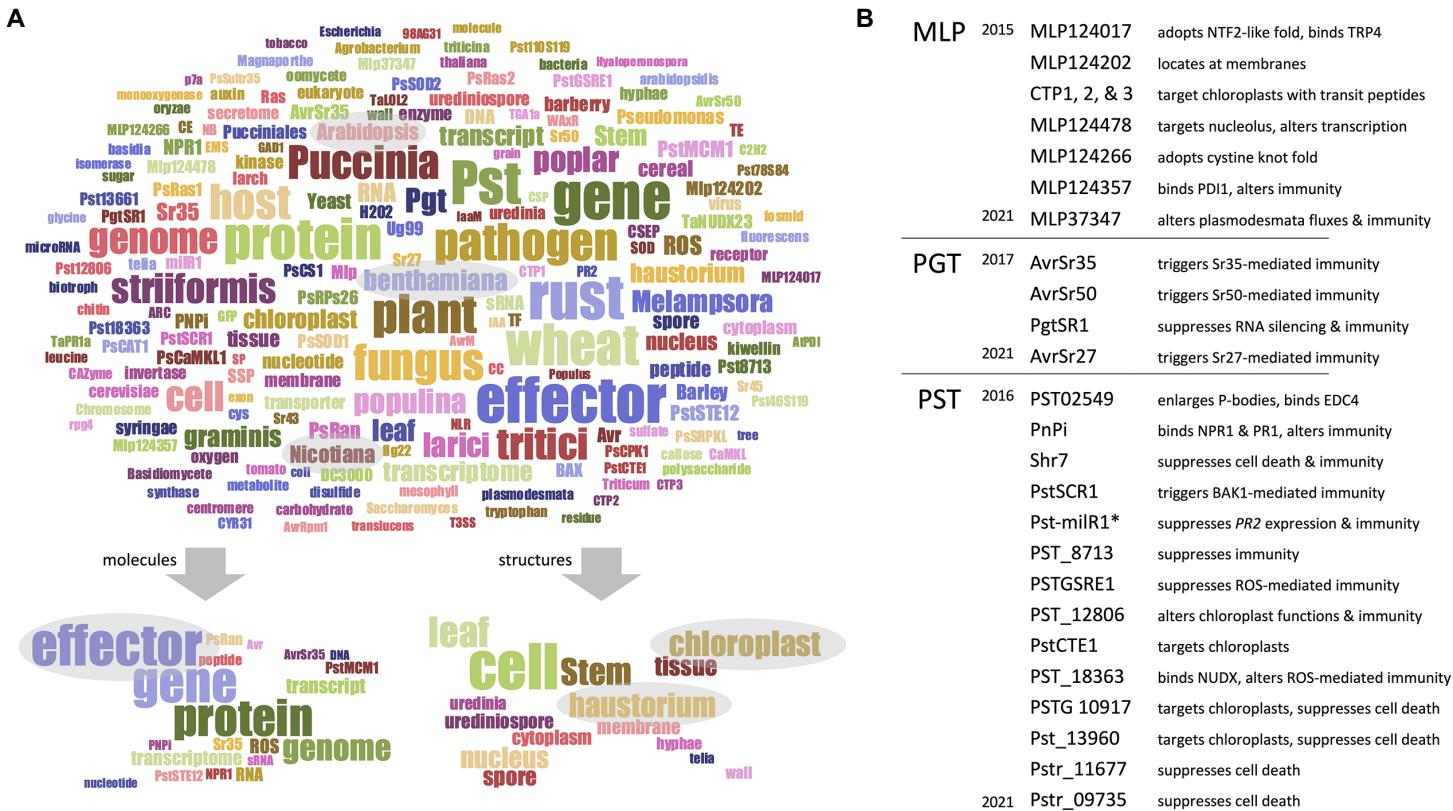
Pucciniales genomes facilitated the functional characterization of effector proteins. **(A)** Upper panel – word cloud displaying the most frequently appearing words referring to group of – or individual – molecules, organisms, and structures. The word cloud was built by using a filtered text file comprising RPGs title and abstract deprived from words not explicitly referring to molecules, organisms, or structures. The word cloud displays 200 words; the size of the words positively correlates with their frequency in the text file. Word colors are meaningless; they simply assist reader visual word discrimination. **(A)** Lower panel – subordinate word clouds highlighting the words referring to molecules (leaf-hand side) or structures (right-hand side). Gray ovals emphasize words discussed in the main text. **(B)** List of effector proteins collectively investigated by the RPGs, grouped according to their rust fungal species, and sorted according to the date of publication of the corresponding RPG reporting their characterization. MLP, *Melampsora larici-populina*; PGT, *Puccinia graminis* f. sp. *tritici*; PST, *Puccinia striiformis* f. sp. *tritici*. The asterisk indicates the non-proteinaceous, small RNA effector Pst-milR1. A short summary accompanies each effector, to inform on the main findings obtained by the functional studies. The raw data used to build this figure are available in Supplementary Dataset 1 (columns Q–W) and Supplementary Dataset 2.

Academic research often addresses so-called “objects,” which can notably be individual (or groups of) organisms (e.g., wheat, fungi, and pathogens), molecules (e.g., proteins, mRNA, and effectors), or structures (e.g., leaves, cells, haustoria, and chloroplasts). To snapshot the objects mainly addressed by RPGs, we performed a word occurrence analysis of RPG titles and abstracts, as described before (Supplementary Dataset 2) (Petre et al., 2022). This analysis generated a word cloud of 200 frequently occurring words (Figure 2A). Firstly, regarding the organisms, words referring to rust species or to their host (e.g., “rust,” “*Puccinia*,” “*Melampsora*,” “pathogen,” “host,” “wheat,” “poplar,” and “plant”) dominated the word cloud, along with words referring to the two model plants *Arabidopsis thaliana* or *Nicotiana benthamiana*, which are not host to pucciniales, but serve extensively as heterologous systems (Lorrain et al., 2018).

Secondly, regarding the molecules, words referring to genomes, genes, proteins, and effectors dominated the word cloud. Notably, the word effector appears in 35%, 60%, and 91% of RPG titles, abstracts, and main texts, respectively; moreover, half of the RPGs from the “functional analysis” category (20 out of 40) mention this word in their title. Also, 72% of the RPGs (59 out of 82) significantly addressed or discussed effectors, and in total 27 effectors were functionally characterized (Supplementary Dataset 1 – columns Q–T; Figure 2B). All the RPGs reporting the characterization of those 27 effectors were released after 2015, 2016, or 2017 for *M. larici-populina*, *P. striiformis* f. sp. *tritici*, and *P. graminis* f. sp. *tritici*, respectively. The functional studies mostly characterized effector features pertaining to their (i) subcellular localization in plant cells (e.g., nucleus, membranes, and chloroplasts), (ii) plant protein interactors (e.g., immune receptors, candidate virulence targets), (iii) ability to alter plant cell physiology (e.g., alteration of sub-cellular structures and fluxes), (iv) ability to trigger or suppress immunity (e.g., recognition by immune receptors, elicitation or suppression of cell death or immunity), and (v) tridimensional structure (e.g., cystine fold) (Figure 2B). Altogether, this shows that the most recent RPGs predominantly focus on effector functional characterization, suggesting that genome availability directly facilitated those functional studies.

Finally, regarding the structures, words referring to cellular structures central for the infection process (e.g., cells, haustoria) dominated the word cloud (Figure 2A). That was expected as haustoria are key infection structures that invaginate host cells, which have been, and still are, highly investigated in plant pathology (Hahn and Mendgen, 1997; Garnica et al., 2014; Bozkurt and Kamoun, 2020). Interestingly, the word chloroplast also appears frequently, as eight RPGs collectively reported 11 candidate effector proteins that target chloroplasts (Petre et al., 2015, 2016a,b; Germain et al., 2018; Xu et al., 2019; Andac et al., 2020; Ozketen et al., 2020; Jaswal et al., 2021) (Supplementary Dataset 1 – columns U–W). Chloroplasts emerge as important actors of plant immunity, and the list of pathogen effectors that target chloroplast rapidly expands (Kretschmer et al., 2019; Gao et al., 2020; Littlejohn et al., 2021; Savage et al., 2021). As obligate biotrophs that intimately associate with their hosts, rust fungi probably need to thoroughly manipulate chloroplast functions; future studies may reveal how effector proteins assist such manipulation.

## Progresses made in other rust species

Early rust genomics achievements assisted the sequencing of the genomes or of the transcriptome of other rust species, opening avenues for comparative genomics or comparative transcriptomic studies (Duplessis et al., 2013; Nemri et al., 2014; Pendleton et al., 2014; Cuomo et al., 2017; Tao et al., 2017, 2019; Chen et al., 2019). Indeed, the genomes of nearly a dozen pucciniales species are now publicly available, and molecular resources have been compiled and made available for the community (Figueroa et al., 2020; Adams et al., 2021). In addition, progresses made with the different generations of sequencing technologies have facilitated ambitious rust sequencing projects. Indeed, the large size and the high repeat content of some rust fungal genomes has precluded their sequencing and assembly (Carvalho et al., 2014; Loehrer et al., 2014). Only recently, sequencing and assembly of larger rust genomes (of hundreds of Megabases to over a Gigabase) was made possible by including high amounts of long molecule sequencing, for instance by using the PacBio HiFi technology (Wu et al., 2020; Tobias et al., 2021; Henningsen et al., 2022; Liang et al., 2022). Notably, the recent genome analysis of three isolates of the Asian soybean rust fungus *Phakopsora pachyrhizi* revealed extremely high (>90%) content of transposable elements and a drastic expansion of gene families related to amino acid metabolism (Gupta et al., 2022). Finally, for a handful of rust species, such as *P. pachyrhizi*, *Hemileia vastatrix* (the coffee rust fungus), or *Puccinia triticina* (the wheat leaf rust fungus), coordinated studies involving genomics, transcriptomics, and gene family analyses have revealed candidate effectors; some of which have been already functionally investigated (Kunjeti et al., 2016; Qi et al., 2016, 2018; Segovia et al., 2016; Maia et al., 2017).

## Towards fully phased genomes and a better understanding of pucciniales-specific genes

Overall, considering the complexity of rust fungal genomes, it took about 10 years to reach a point to which haplophased genome sequencing from dikaryotic spores was possible with all chromosomes assembled telomere to telomere. Improvement of long read sequencing technologies such as PacBio or Nanopore and emergence of methods allowing chromosome conformation capture such as Hi-C were pivotal to overcome all assembly troubles related to the highly repeated profile of large rust genomes (Duan et al., 2022). Despite the different pipelines used by different consortia to annotate first-generation rust genomes and the heterogeneity they may have generated, they have allowed robust comparative or population genomics studies (Aime et al., 2017; Lorrain et al., 2019). With more systematic efforts to reach complete haplophased rust genomes, the community will be soon able to draw deeper and even more robust comparative analyses to delineate the evolution of gene or transposable element families. Another remarkable feature of rust fungi genomes is the amount of genes of unknown function, which are restricted to the order Pucciniales (Aime et al., 2017). Although a fraction of these genes may be wrongly predicted, they might reflect the paucity of knowledge of the rust biology and of their particularly complex life cycles (Duplessis et al., 2021). A particularly appealing and challenging goal in the future of rust biology research will be to assign functions to these genes. The past decade has been marked by the emergence of machine-learning driven prediction tools to identify effectors or protein sub-cellular localization, such as EffectorP, Localizer, or ApoplastP (Sperschneider et al., 2017a,b; Sperschneider and Dodds, 2022). Improvement of such bioinformatic-assisted methods will be instrumental to decipher the largely unexplored genomic landscape of the pucciniales.

## Conclusion and outlook

Over the last decade, post-genomics approaches have enabled the study of model rust fungi, and particularly the functional characterization a specific proteins. Current research efforts simultaneously improve genome sequence qualities and enlarge the list of rust species investigated; both facilitating comparative studies of rust fungi. In the future, such studies may tackle key questions such as “to what extent do rust fungi manipulate hosts chloroplasts?”, “how do rust fungi regulate their complex life cycle?”, or “how do rust fungi evolved to adapt to specific host plants?”

## Author contributions

BP and SD: conception and design of the study, and drafting, revising, and editing of the manuscript. BP: data acquisition, analysis, and interpretation. All authors contributed to the article and approved the submitted version.

## Funding

The authors were supported by grants overseen by the French PIA Programmes Lab of Excellence ARBRE (ANR-11-LABX-0002-01), Pôle Scientifique Agronomie, Agroalimentaire, Forêt – Université de Lorraine, and Région Grand Est (France).

## Conflict of interest

The authors declare that the research was conducted in the absence of any commercial or financial relationships that could be construed as a potential conflict of interest.

The handling editor declared a shared affiliation with the author SD at the time of review.

## Publisher’s note

All claims expressed in this article are solely those of the authors and do not necessarily represent those of their affiliated organizations, or those of the publisher, the editors and the reviewers. Any product that may be evaluated in this article, or claim that may be made by its manufacturer, is not guaranteed or endorsed by the publisher.
